# Pseudovirus as an Emerging Reference Material in Molecular Diagnostics: Advancement and Perspective

**DOI:** 10.3390/cimb47080596

**Published:** 2025-07-29

**Authors:** Leiqi Zheng, Sihong Xu

**Affiliations:** 1 Division II of In Vitro Diagnostics for Infectious Diseases, Institute for In Vitro Diagnostics Control, National Institutes for Food and Drug Control, Beijing 100050, China; 13051670291@163.com; 2 State Key Laboratory of Drug Regulatory Science, Beijing 100050, China; 3 NMPA Key Laboratory for Quality Research and Evaluation of Medical Devices, Beijing 100050, China; 4 NMPA Key Laboratory for Quality Research and Evaluation of In Vitro Diagnostics, Beijing 100050, China

**Keywords:** RNA virus, nucleic acid testing, reference material, pseudovirus

## Abstract

In recent years, the persistent emergence of novel infectious pathogens (epitomized by the global coronavirus disease-2019 (COVID-2019) pandemic caused by severe acute respiratory syndrome coronavirus 2 (SARS-CoV-2)) has propelled nucleic acid testing (NAT) into an unprecedented phase of rapid development. As a key technology in modern molecular diagnostics, NAT achieves precise pathogen identification through specific nucleic acid sequence recognition, establishing itself as an indispensable diagnostic tool across diverse scenarios, including public health surveillance, clinical decision-making, and food safety control. The reliability of NAT systems fundamentally depends on reference materials (RMs) that authentically mimic the biological characteristics of natural viruses. This critical requirement reveals significant limitations of current RMs in the NAT area: naked nucleic acids lack the structural authenticity of viral particles and exhibit restricted applicability due to stability deficiencies, while inactivated viruses have biosafety risks and inter-batch heterogeneity. Notably, pseudovirus has emerged as a novel RM that integrates non-replicative viral vectors with target nucleic acid sequences. Demonstrating superior performance in mimicking authentic viral structure, biosafety, and stability compared to conventional RMs, the pseudovirus has garnered substantial attention. In this comprehensive review, we critically summarize the engineering strategies of pseudovirus platforms and their emerging role in ensuring the reliability of NAT systems. We also discuss future prospects for standardized pseudovirus RMs, addressing key challenges in scalability, stability, and clinical validation, aiming to provide guidance for optimizing pseudovirus design and practical implementation, thereby facilitating the continuous improvement and innovation of NAT technologies.

## 1. Introduction

Highly transmissible and pathogenic RNA viruses, such as severe acute respiratory syndrome coronavirus 2 (SARS-CoV-2), Ebola virus (EBOV), respiratory syncytial virus (RSV), and Middle East respiratory syndrome virus (MERS-CoV), continue to instigate regional and global public health crises. Outbreaks caused by these pathogens not only impose substantial disease burdens but also threaten societal stability through secondary effects, including healthcare resource depletion and economic stagnation. Under this background, establishing rapid and accurate pathogen detection systems has emerged as a critical requirement for timely interrupting disease transmission chains and implementing precise containment measures. Conventional pathogen detection techniques—such as microbial cultivation, hemagglutinatia inhibition, and enzyme-linked immunosorbent assays (ELISA) are hampered by inherent shortcomings such as long detection time, suboptimal specificity/sensitivity, poor automation compatibility, and limited throughput, rendering them insufficient for rapid and accurate diagnosis. Breakthroughs in molecular diagnostics have addressed these challenges: nucleic acid testing (NAT) technologies targeting conserved pathogenic sequences enable early-stage identification of pathogens through specific nucleotide sequence recognition, thereby facilitating timely intervention and treatment. This capability proves crucial for controlling the transmission and epidemic spread of infectious pathogens. Notably, during the SARS-CoV-2 pandemic, the global utilization of NAT surged exponentially. Through critical applications, such as confirmed case triage and viral variant tracking, NAT significantly reduced severe case incidence and transmission coefficients, establishing itself as a pivotal defense strategy in pandemic control. To ensure the reliability of NAT results, standardization and quality control of experimental reagents and operational procedures are indispensable. Consequently, regulatory agencies worldwide have progressively established nucleic acid reference material (naRM) repositories and external quality assessment (EQA) programs, with metrological traceability of NAT quantitative values becoming a focal point in molecular diagnostics.

The majority of human pathogenic viruses are RNA viruses, whose high mutation rates and low viral loads pose dual challenges for NAT applications. The NAT procedure of RNA viruses involves five critical steps: sample collection, nucleic acid extraction, reverse transcription, polymerase chain reaction (PCR) amplification, and result analysis. Among these, the RNA extraction and PCR amplification efficiency most substantially influence the accuracy of final results, as suboptimal efficiency in either process may lead to false-negative results. Furthermore, multiple variables, including performance disparities among different commercial kits (attributed to differences in enzyme activity and reaction buffer formulations), equipment-dependent differences in nucleic acid extractors and PCR cyclers, laboratory environments, and operator techniques, collectively influence the reliability of NAT results, underscoring the urgent need for standardized reference materials (RMs).

RMs are substances with sufficiently homogeneous and stable characteristics that are rigorously validated for calibration and verification of measurement systems [1]. Both the World Health Organization’s (WHO) Prequalification of in vitro Diagnostic Assessment and China’s Regulations for the Registration of in vitro Diagnostic Reagents (2021) mandate that diagnostic reagent development must achieve metrological traceability through international/national RMs [2,3]. For RNA virus nucleic acid RMs (naRMs), the international metrology community has achieved a great milestone. Under WHO coordination, the UK National Institute for Biological Standards and Control (NIBSC) established the first international RM for hepatitis C virus (HCV) RNA (NIBSC code 96/790) in 1997, enabling cross-laboratory and cross-methodological comparability of global detection results [4]. In 2016, the National Institute of Metrology (NIM), China pioneered the development of China’s first HCV nucleic acid national Certified RM (GBW(E)090032), which established metrological traceability to the WHO international standard, effectively bridging the critical gap in RNA naRM availability within China’s diagnostic framework, and positioning China as an important global provider of viral nucleic acid certified RMs (CRMs) compliant with International Organization for Standardization (ISO) 17511:2020 requirements [5]. The coronavirus disease-2019 (COVID-2019) pandemic has accelerated the advancement of NAT technologies while simultaneously elevating quality control requirements. Consequently, research and application of naRMs have gained increasing global attention from metrological institutions, international academic organizations, and commercial entities, emerging as a focal area in NAT development. This review systematically examines the classification and characteristics of naRMs, the construction technologies of pseudoviruses based on different packaging systems, and their current applications in NAT development. Additionally, we prospectively analyze the future potential of pseudovirus-based technologies in advancing NAT standardization.

## 2. Types and Characteristics of naRMs

Categories of naRMs include inactivated virus, naked DNA (genomic DNA or plasmid DNA), naked RNA (genomic RNA, in vitro-transcribed RNA, or chemically synthesized RNA fragments), and pseudovirus [6]. The following Section 2.1, Section 2.2 and Section 2.3 provide a detailed introduction to these distinct forms of RMs.

### 2.1. Inactivated Virus

RMs of inactivated viruses manufactured via pathogen isolation and cultivation simulate the biological matrix features of natural infection specimens, thereby establishing traceability benchmarks that most closely mimic authentic clinical scenarios for NAT [7]. Their key advantage lies in preserving the intact viral nucleocapsid-nucleic acid complex architecture, enabling systematic validation of critical experimental parameters, including viral lysis efficiency and inhibitor tolerance ability [8]. However, their development and application face multiple technical challenges: (i) limited production scalability due to sample scarcity and heterogeneity caused by residual host cell proteins, complicating inter-batch quality control [9,10]; (ii) high biosafety risks requiring containment facilities with bio-safety level (BSL)-3/4 certification, particularly for highly pathogenic viruses, which restricts the number of laboratories that can participate, thus impeding research and production progress [11]; (iii) thermal inactivation, while eliminating viral pathogenicity, may concurrently induce structural damage to virions and nucleic acid fragmentation, potentially compromising the measurement accuracy [12]; (iv) the methodology imposes stringent technical expertise requirements: the inactivation process necessitates meticulous optimization to balance biosafety imperatives with preservation of native virion architecture and genomic integrity, demanding advanced operator proficiency in viral biophysics.

### 2.2. Naked DNA/RNA

RMs of naked DNA or RNA, predominantly recombinant plasmid DNA and in vitro-transcribed RNA, display many advantages: simplified designation and preparation, rapid generation rate, high quantification precision, and large-scale batch production. Their quality control is readily achievable through standardized manufacturing and validation protocols. However, the simplified architecture of these naked nucleotides necessitate critical evaluation when they were used as RM in NAT: (i) the absence of viral capsid-nucleic acid complexes precludes assessment of clinical matrix effects during nucleic acid extraction, while simplified nucleotide architectures (e.g., plasmid vs. authentic virion RNA) result in significant differences in nucleic acid quantification efficiency compared to real samples; (ii) process efficiency losses in viral lysis and nucleic acid purification remain unaddressed; (iii) stability challenges persist, particularly for RNA RMs, which face degradation risks from ubiquitous RNases and intrinsic instability during preparation, aliquoting, storage, and transport [6,13].

### 2.3. Pseudovirus

Pseudoviruses, chimeric recombinant viral particles combining nucleic acids and envelope proteins from distinct viruses, represent an innovative solution. Through targeted genomic modifications (e.g., deletion of envelope protein-coding genes), researchers can precisely abrogate autonomous replication capacity and progeny proliferation features [14]. This engineering strategy minimizes biosafety risks, establishing pseudoviruses as ideal surrogates for virological studies [15]. Current applications of pseudoviruses span vaccine efficacy evaluation, viral entry mechanism elucidation, and NAT kit quality control [15,16,17]. As naRMs, pseudoviruses exhibit five core advantages: (i) whole NAT process parallelism: full participation in viral lysis, nucleic acid extraction, reverse transcription, amplification, and detection workflows, mirroring authentic clinical sample behaviors; (ii) enhanced stability: nucleocapsid protection against enzymatic degradation facilitates long-term storage and transportation; (iii) error mitigation: concurrent processing with clinical samples enables monitoring of extraction efficiency, reagent/instrument performance, and amplification fidelity, reducing false-positive/negative outcomes; (iv) scalable production: high-yield pseudoviruses can be obtained via cellular transfection by using optimized procedures; (v) BSL-2 compatibility: single-cycle infectivity eliminates biosafety concerns, enabling widespread NAT applications [18,19,20,21,22].

## 3. Technologies of Pseudovirus Construction

Pseudovirus-based RMs refer to standardized pseudoviral products engineered for virological study, vaccine research, and NAT assays, ensuring experimental consistency and reproducibility. Current construction methodologies for pseudoviruses are primarily categorized based on the types of backbone virus, with classification criteria simplified as follows: (i) nucleic acid type: DNA pseudoviruses (e.g., adenoviral vectors) versus RNA pseudoviruses (e.g., lentiviral vectors); (ii) structural features: enveloped pseudoviruses (e.g., vesicular stomatitis virus-based systems) versus non-enveloped pseudoviruses (e.g., MS2 bacteriophage-based systems). Notably, significant disparities exist in assembly efficiency and target compatibility across different pseudoviral packaging systems, necessitating scenario-specific optimization. Types of pseudoviral packaging systems and their applications were summarized in Table 1. A detailed introduction to these systems is provided in subsequent sections.

### 3.1. Lentiviral Vector Packaging Systems

Lentiviral vectors have emerged as the predominant platform for pseudovirus preparation due to their high efficiency transduction of non-dividing cells and stable genomic integration. Viruses of lentiviral vector packaging systems include those derived from human immunodeficiency virus type 1 (HIV-1), simian immunodeficiency virus (SIV), and feline immunodeficiency virus (FIV). Using the HIV-1 system as a paradigm, the split plasmid design principle (typically partitioning into 2–4 discrete plasmids: e.g., packaging, transfer, and envelope-expressing plasmids) enables lentiviral vectors to achieve modular control over Gag/Pol assembly, transgene delivery, and heterologous envelope protein expression. This is accomplished through physical segregation of viral replication essential components (e.g., reverse transcriptase genes and envelope glycoprotein (Env) genes), coupled with tissue-specific promoters (e.g., cytomegalovirus (CMV) promoter-driven Env expression). The strategic configuration preserves pseudoviral surface antigen functionality while reducing the recombination risks to <0.001% [23,24]. To address biosafety concerns associated with HIV-1, the SIV and FIV systems have been developed. For example, the SIVmac25 system has been utilized for constructing SARS-CoV-1 pseudoviruses for antiviral drug screening. Meanwhile, the FIV system demonstrates superior viral titers (from 1.5 to 2.3-fold higher than using the HIV-1 system) in pseudovirus production for pathogens such as EBOV and dengue virus (DENV) [25,26].

### 3.2. Vesicular Stomatitis Virus Packaging System

Vesicular stomatitis virus (VSV), an enveloped negative-strand RNA virus (genome length ~11 kb), serves as an ideal pseudoviral vector owing to its low human pathogenicity, broad mammalian cell tropism, and rapid replication kinetics (reaches peak within 8–12 h post-infection) [27,28]. The foundational work by Stillman et al. in 1995, which rescued the recombinant virus in vitro by cloning the VSV genomic sequences into plasmids using reverse genetics, enabled subsequent engineering [29]. Notably, VSV exhibits exceptional compatibility with heterologous envelope proteins (e.g., EBOV glycoprotein), facilitating the construction of target-specific pseudoviruses for studying virus–host interactions and screening antiviral compounds [30,31,32,33]. Furthermore, replacing the VSV G glycoprotein gene with reporter genes (e.g., fluorescent proteins, luciferase, or secreted alkaline phosphatase) allows real-time quantification and tracking of pseudoviral infectivity [34,35].

### 3.3. Retroviral Packaging System

Retroviral packaging system, also called murine leukemia virus (MLV) packaging system, based on moloney MLV (MMLV, ~8.2 kb positive-sense RNA virus), achieving replication incompetence by deletion of critical elements such as the packaging signal (Ψ) and long terminal repeats (LTR) [36]. The strategy for constructing MMLV pseudoviruses involves the following: firstly, integrating Gag-Pol structural genes into Ψ/LTR-deficient plasmid to generate stable packaging cell lines, and secondly, co-transfecting these cells with an Env (heterologous virus envelope protein)-expression plasmid and a target gene transfer plasmid to yield recombinant retroviruses [36,37]. This system has been extensively applied in mechanistic research of EBOV, MERS-CoV, and SARS-CoV-2 infection, as well as antibody neutralization studies [23].

### 3.4. Adenovirus and Adeno-Associated Virus Packaging Systems

The development of adenovirus (AdV) and adeno-associated virus (AAV) vectors represents a significant advancement in gene therapy, with both systems exhibiting unique advantages for gene delivery and therapeutic applications. AdV, a non-enveloped double-strand DNA virus (genome length ~36 kb), has emerged as a cornerstone tool in gene therapy due to its broad cellular tropism (spanning both dividing and non-dividing cells) and modular genomic architecture. AdV5-derived vectors (e.g., AdEasy/AdMAX systems) achieve highly efficient exogenous gene delivery (capacity up to 8 kb) through the deletion of pathogenic genes (e.g., E1/E3) while retaining capsid-targeting functionality [38]. Their versatile administration routes (intravenous, respiratory aerosolization, and oral delivery) and scalable production procedures have facilitated clinical applications in diverse areas, ranging from tumor immunotherapy (e.g., oncolytic virus engineering) to vaccine development [38]. AAV, classified as a non-enveloped single-stranded linear DNA virus, is recognized as one of the structurally simplest replication-defective parvoviruses. The non-pathogenic feature of AAV ensures enhanced safety in clinical applications, establishing it as the safest viral vector currently. As a replication-incompetent virus, AAV requires co-infection with helper viruses (e.g., AdV or herpesvirus) for productive replication and host cell lysis. These characteristics, coupled with sustained transgene expression and low immunogenicity, position AAV as a highly promising gene transfer vehicle, which is widely utilized in gene therapy and vaccine research [39,40].

### 3.5. Armored RNA Packaging System

Armored RNA, a pseudoviral technology developed by Pasloske et al. in 1997, involves the encapsulation of recombinant RNA within MS2 bacteriophage capsid proteins. This innovative approach was specifically engineered to address the challenges in preparing standardized RNA quality control materials for NAT assays, establishing a robust platform for RNA stability and quantification traceability [41,42]. The principle involves employing genetic engineering techniques to clone the gene sequence encoding the *Escherichia coli* (*E. coli*) MS2 bacteriophage capsid proteins and exogenous target sequences into an expression vector. The recombinant plasmid is then transformed into *E. coli* for induced expression. During this process, the exogenous gene is transcribed into RNA, which is encapsulated within the MS2 capsid proteins. These components are ultimately assembled into an icosahedral RNA–protein complex, termed armored RNA or MS2 virus-like particles (VLPs) [43]. This innovative armored RNA technology addresses the critical challenge of naked RNA degradation during sample processing by generating standardized quality control materials that faithfully simulate the nucleic acid extraction procedure of authentic viruses. Consequently, it has gained widespread application in molecular diagnostics for nucleic acid detection and quality assurance [43,44,45].

### 3.6. Other Packaging Systems

Recent advances in molecular virology have witnessed the emergence of reverse genetics-based technologies for engineering self-replicating RNA viruses. These methodologies enable precise manipulation of RNA viruses at the cDNA level through site-directed mutagenesis, insertions, substitutions, deletions, and genomic rearrangements to construct pseudoviruses. The foundational breakthrough occurred in 1978 when Taniguchi et al. established the first reverse genetics system for a positive-sense, non-segmented RNA virus, achieving the in vitro rescue of the Q_β_ bacteriophage—a milestone that promoted subsequent reverse genetics development [46]. Subsequently, with advancements in reverse genetics methodologies, scientists extended these techniques to rescue negative-strand RNA viruses, leading to the establishment of multiple reverse genetics platforms. Contemporary developments have yielded diverse self-replicating RNA viral vectors (RNA replicon vectors), including alphavirus-, flavivirus-, picornavirus-, and paramyxovirus-derived systems. These engineered vectors have been validated as recombinant VLPs capable of mediating efficient gene delivery, facilitating exogenous antigen expression, and subsequently eliciting robust host immune responses. They have been extensively employed not only in fundamental virological investigations, such as elucidating molecular mechanisms underlying viral replication and translation, but also in applied research areas including the study of novel vaccines and development of high-throughput antiviral drug screening systems [47,48,49].

## 4. Advantages and Challenges of Pseudoviruses as RMs in NAT

Pseudoviruses play an indispensable role as naRMs in the detection of infectious pathogens, such as SARS-CoV-2, crucially supporting the standardization, accuracy, and reliability of NAT assays. The following sections provide a comprehensive analysis of both the operational advantages and current technical limitations in their application as naRMs.

### 4.1. Advantages of Pseudoviruses as naRMs

Most zoonotic viruses causing human diseases are enveloped RNA viruses. Pseudovirus-based RMs are engineered as chimeric recombinant viral particles comprising an envelope protein encapsulating inner nucleic acids. These materials undergo rigorous standardization to ensure experimental consistency and reproducibility [12]. The unique advantages of pseudoviruses as RMs in molecular diagnostics are elaborated below.

**Figure 1:**
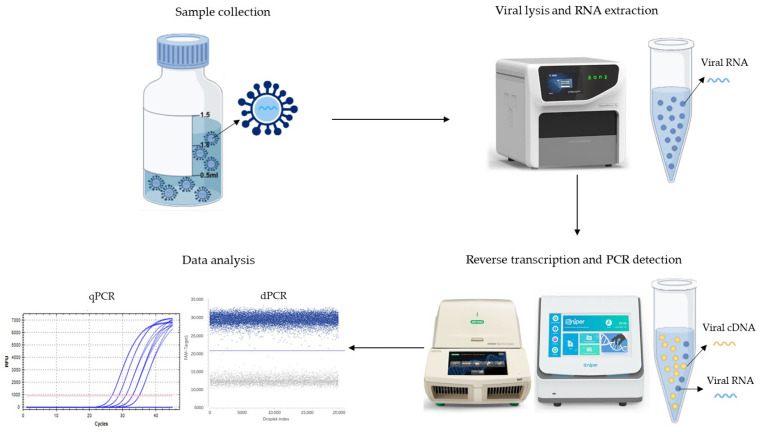
Entire workflow of NAT process from sample preparation to data interpretation.

Providing standardized analogs mimicking native viruses. Ideal naRMs should demonstrate full compatibility with the clinical specimen processing procedure to ensure comprehensive quality control throughout the entire workflow from sample preparation to data interpretation (Figure 1). While naked nucleic acids are restricted to amplification process validation, inactivated viruses face practical limitations due to source scarcity and persistent biosafety concerns. Pseudoviruses feature precisely designed architectures where encapsulated RNA within protein capsids accurately simulates the kinetic profile of viral genome release observed in clinical specimens. This structural fidelity ensures reliable performance across critical procedural stages, including RNA extraction, reverse transcription, amplification, and quantitative analysis, thereby significantly enhancing the metrological traceability and diagnostic accuracy of molecular testing [10,12].

Enabling systematic evaluation of reagents and instruments. The cost-effectiveness and production scalability of pseudoviruses facilitate comprehensive multi-parametric evaluation of NAT technology, including nucleic acid extraction efficiency, limits of detection (LoD), precision, stability, and interference resistance, facilitating evidence-based optimization of molecular detection platforms. For instance, Yan et al. in 2021 utilized lentiviral-based SARS-CoV-2 pseudoviruses to evaluate seven commercial NAT kits, demonstrating good concordance between pseudovirus and SARS-CoV-2 cell culture supernatant in final outcomes [50]. This investigation not only established quantitative correlations between pseudovirus and clinical specimens but also developed an evaluation system integrating multiple parameters to objectively analyze inter-kit performance. These findings collectively prove that pseudovirus-based naRMs can provide a reliable technical pathway for the selection and optimization of clinical diagnostic tools.

Enhancing sensitivity, specificity, and repeatability. Serving as naRMs, pseudoviruses can closely mimic the behavior of authentic viruses in experimental settings and can be integrated throughout the entire NAT workflow, enabling researchers to systematically identify and optimize each step of the detection process. This approach helps eliminate false-positive/false-negative outcomes while improving detection specificity. Additionally, pseudoviruses facilitate the optimization of detection sensitivity, ensuring reliable results even at low viral concentration, thereby enhancing the overall performance of NAT [50,51]. Furthermore, the intrinsic stability of pseudoviruses allows researchers to conduct experiments under identical conditions across different laboratories, effectively validating result reliability and experimental repeatability [52].

Accelerating technological innovation in pathogen molecular detection. Pseudoviruses carrying signature nucleic acid sequences serve as important platforms for advancing diagnostic technologies. Point-of-care testing (POCT), as an example, integrates nucleic acid extraction, amplification, and detection into a single device to achieve fully automated processing and analytical steps, thereby enabling direct and rapid delivery of reliable results. The envelope-encapsulated structure of pseudoviruses ensures tolerance to diverse detection environments, guaranteeing accurate performance validation in automated systems. These attributes position the pseudovirus as a reliable validation model for emerging technologies, significantly accelerating the translational application of novel diagnostic platforms.

Facilitating global collaboration and harmonization. The unique structural advantages of pseudoviruses confer exceptional stability during international transportation. Additionally, their single-cycle infection property inherently eliminates biosafety risks at the molecular design stage, fully complying with stringent international logistics regulations. These characteristics enhance the global accessibility and deployment of pseudovirus-based naRMs, driving organizations like the WHO to establish global platforms for standardized NAT performance validation. By sharing standardized pseudoviral materials and harmonizing quality control criteria, laboratories worldwide achieve direct inter-laboratory data comparability, effectively resolving discrepancies caused by heterogeneous materials [53]. This collaboration not only expedites the validation pipeline for emerging pathogen detection technologies but also provides the technical infrastructure for real-time surveillance of pathogen evolution and the construction of a worldwide pandemic alert network. Collectively, these advances promote the transformation of infectious disease management from fragmented research paradigms to systematic, artificial intelligence (AI)-enhanced global defense frameworks.

### 4.2. Challenges in the Construction and Application of Pseudovirus-Based naRMs

While pseudoviruses become an indispensable tool for quality control in NAT, their development and practical implementation confront persistent challenges. These include optimization of productive efficiency and yield, inherent complexities in design/exploitation, standardization of quantification methodologies, batch-to-batch homogeneity, long-term stability under diverse storage conditions, biosafety compliance problems, and technical/infrastructural requirements for large-scale deployment. The subsequent sections will elaborate on these challenging technical issues.

Productive efficiency and yield limitations. Scalable production of high-titer pseudoviruses remains a primary technical bottleneck. Achieving optimal yield requires precise tuning of multiple parameters, such as packaging systems modification, expression vector configurations, host cell line selection, transfection protocols optimization (e.g., plasmid/reagent ratios), post-transfection harvesting windows, and virion enrichment methods [23].

Design complexity and pathogen-specific optimization. Unlike pseudotyped particles employed in vaccine/drug evaluation, pseudoviruses applied in the NAT area should precisely simulate the physical encapsulation states of native viral genomes. The envelope architecture not only functions as a nucleic acid protective shell but also directly affects lysis reagent permeability and nucleic acid extraction efficiency. For instance, comparative studies reveal that discrepancies in SARS-CoV-2 virion mimicry between lentiviral and MS2 bacteriophage packaging systems can induce up to 40% variability in RNA extraction efficiency, underscoring the importance of packaging system selection [50]. The optimal packaging system may substantially differ when constructing pseudoviruses for different pathogens or even when targeting alternate genomic loci within the same virus. Furthermore, certain pseudovirus production workflows exhibit stringent host cell dependencies. All of these necessitate case-specific selection of packaging systems, backbone plasmids, and permissive cell lines [12,23,50]. However, current mainstream packaging systems (e.g., lentiviral platform, MS2 bacteriophage platform) lack universal compatibility, highlighting the need for novel packaging platforms with broader pathogen adaptability.

Standardization challenges of quantification methodologies. As naRMs, pseudoviruses are typically quantified using copy number concentration (e.g., copies/mL) rather than virion counts. The metrological conversion from viral particles to nucleic acid equivalents must navigate three technical barriers: (i) RNA extraction efficiency, (ii) reverse transcription yield, and (iii) quantitative PCR (qPCR) or digital PCR (dPCR) quantification accuracy. Critical variability arises at each stage—commercial RNA extraction kits exhibit < 100% recovery rates with significant inter-kit variability (Coefficient of variation (CV): 18–35%), while reverse transcription efficiencies rarely exceed 70% across different enzyme systems. Inter-platform discrepancies in qPCR or dPCR quantification further compound these challenges. Recent multi-platform evaluations revealed 2.3 log variance in measured copy numbers across different dPCR platforms when analyzing identical pseudovirus samples, which is primarily attributable to microfluidic droplet heterogeneity and fluorescence threshold inconsistencies [54]. Additionally, environmental factors (e.g., aerosol contamination rates, thermal cycler ramp speeds) and operator-dependent variables further introduce inter-laboratory variability, potentially yielding discordant quantification results under different experimental conditions. Addressing these issues requires harmonized protocols and robust statistical frameworks to ensure metrological traceability and cross-laboratory comparability.

Plasmid DNA clearance and residual ratio control. During pseudovirus assembly, plasmid DNA carrying target sequences is transfected into host cells, which enables intracellular virion packaging followed by secretion into the cell culture supernatant. Going through subsequent processing steps, including filtration, purification, centrifugation, and enrichment, to yield the final pseudoviral solution. The inherent stability of plasmid DNA poses a critical challenge: residual plasmid persisting through the production process may co-purify with viral nucleic acids during extraction. This contamination greatly impacts quantification validity, as plasmid-borne viral sequences can serve as unintended templates in PCR-based assays, generating systematic quantification bias, even false-positive results. Mitigation strategies include (i) optimizing plasmid-to-reagent ratios to ensure transfection efficiency while minimizing residual DNA; (ii) using phosphate-buffered saline (PBS) washes post-transfection (usually the day after transfection) to remove unbound plasmids; (iii) adding DNaseI during virion purification and RNA extraction to degrade surface-adherent or free plasmid DNA [55,56,57]. What is more, the residual plasmid level in final products must be rigorously quantified via qPCR/dPCR to ensure compliance with safety thresholds.

Homogeneity and stability assurance. Batch-to-batch consistency and long-term stability are critical for RM certification. Manufacturers must conduct intra-/inter-batch homogeneity assessments to verify uniform physicochemical properties of RMs. Additionally, considering practical application scenarios, optimal storage matrices and conditions (e.g., lyophilization vs. liquid) require systematic investigation to enhance pseudovirus stability during routine transport (e.g., dry ice) and ordinary laboratory storage conditions (storage at 2–8 °C or −20 °C).

Biosafety management and regulatory compliance. Implementation of risk-proportionate containment strategies must be mandated across all pseudovirus production and application stages, requiring rigorous adherence to the WHO Laboratory Biosafety Manual (4th edition) guidelines and Institutional Biosafety Committee (IBC) oversight. While replication-deficient vectors are typically employed, multi-plasmid transfection systems retain latent risks of homologous recombination, potentially restoring viral proliferation competence or generating novel infection pathways [58,59]. Therefore, researchers must ensure laboratory compliance with biosafety standards (operations in BSL-2 or higher containment facilities) while conducting systematic biosafety assessments during both the initial preparation phase and subsequent implementation stages. These evaluations must address potential health risks associated with pseudovirus applications. Enhanced governance and monitoring strategies for standardized pseudoviral RMs are critical to mitigating biosafety risks, thereby establishing robust safeguards for both viral research and public health security.

Technical and financial constraints. The design and generation of pseudoviruses typically require substantial financial investments in technology, equipment, reagents, and personnel resources. Particularly during the generation of high-quality pseudovirus-based naRMs, insufficient funding may impede research progress. Disparities in laboratory resources and technical capabilities further complicate the standardized production and application of pseudoviruses. Identifying funding sources and establishing collaborative partnerships represent the most straightforward and effective strategy to address this challenge.

## 5. Future Directions for Optimized Pseudoviral naRMs and Application Prospects

It is evident that pseudoviruses serve as effective and safe pathogen substitutes in contemporary scientific research, playing an increasingly significant role in both infectious disease research and NAT development. The growing demand for pseudovirus as a novel naRM is prompting the development of a packaging system capable of meeting the requirements of excellent safety, large fragment encapsulation capacity, high yield, broad compatibility, as well as good stability and sustainability. Future optimization of standardized pseudoviral naRMs requires multi-dimensional strategies to improve analytical scientificity and diagnostic reliability, thereby expanding their applications in molecular diagnostics for emerging infectious diseases.

Optimizing pseudovirus packaging systems and developing diversified platforms. Previous studies proved that current systems employed in nucleic acid pseudoviral RM production exhibit inherent limitations and have not always been successful in generating certain types of RNA viruses. To better simulate broader types of pathogens, novel pseudoviral packaging systems must be engineered. Notably, reverse genetics-derived alphaviral systems, flaviviral systems, and picornaviral packaging systems have demonstrated robust exogenous gene transfer capabilities and achieved validated applications in vaccinology studies [23], suggesting their potential as alternative packaging backbones for naRM generation. Future efforts should be focused on integrating molecular biology techniques with virological approaches to develop a broader variety of pseudovirus packaging systems, thereby satisfying the growing demand for standardized naRMs across different pathogen species.

Genomic engineering of viral sequences for enhanced yield and stability of pseudoviruses. In certain scenarios, targeted genomic modifications can improve the titer and stability of pseudoviruses under specific experimental conditions. For instance, codon optimization facilitates the rational engineering of pseudoviral envelope protein sequences, enhancing their expression levels in host cells and thereby increasing pseudoviral yields. Furthermore, many studies have proved that the subcellular localization of envelope proteins critically impacts the assembly and secretion of pseudoviruses. Envelope proteins with plasma membrane localization (e.g., HIV and VSV pseudovirus packaging systems) typically achieve higher viral titers than those whose envelope proteins are retained in organelle membranes (e.g., flavivirus packaging systems) due to inherent organelle retention signals [23,60]. Recent investigations revealed that the subcellular localization of viral envelope proteins is closely associated with their structural features, particularly transmembrane domain organization. Therefore, strategic modifications of original viral sequences, such as removing or replacing organelle retention signals without disrupting protein functionality, truncating cytoplasmic domains, or substituting cytoplasmic regions with sequences from HIV/VSV envelope proteins, have demonstrated potential to enhance pseudovirus packaging efficiency. These optimizations will significantly improve both secretion kinetics and overall pseudoviral yields [23].

Improving safety and controllability in pseudovirus generation and application. Future pseudovirus design should prioritize adaptation to lower biosafety requirements (e.g., BSL-2 laboratories or even routine community clinics) to facilitate broader implementation and utilization, particularly in regions with limited experimental resources. Furthermore, pseudovirus-based naRMs should rigorously mimic the natural structural characteristics of authentic viruses. This fidelity is critical for establishing accurate application models to optimize NAT assays and advance integrated platforms combining nucleic acid extraction with detection modalities, such as POCT systems.

Standardizing the application of pseudovirus-based RMs in NAT. Standardized quantification protocols must be established for the quality assignment of pseudovirus-based naRMs, accompanied by the development of international and national standards. Expanding the repertoire of pseudoviral RMs across diverse pathogens and establishing a standardized repository with certified quantity values are essential to enhance their global utility in NAT workflows.

Enhancing inter-disciplinary collaboration to promote innovation and application of pseudovirus technology. With the advancement of AI and computational biology technologies, there is an opportunity to better utilize large-scale data models for analyzing and optimizing pseudovirus design. This approach enables systematic identification of critical factors influencing pseudoviral yield and characteristics, thereby informing subsequent experimental design. Concurrently, it is imperative to foster inter-disciplinary communication and cooperation among experts in virology, molecular biology, immunology, and related fields. Establishing comprehensive research teams through such cross-disciplinary integration will significantly accelerate technological innovation and practical implementation of pseudovirus research.

## 6. Conclusions

In summary, with the continuous emergence of novel infectious pathogens, pseudoviruses act as a safe and effective detection tool and are poised to play pivotal roles in enhancing diagnostic efficiency, streamlining testing workflows, and advancing technological innovation. It can be anticipated that, along with progressive refinements in pseudovirus platforms, these engineered biological RMs hold the potential for comprehensive deployment across diverse scenarios, ranging from BSL-2 laboratories to community clinics. Such integration could establish a “biological calibration standard” with real-time dynamic calibration capabilities, thereby strengthening global infectious disease monitoring networks.

## Figures and Tables

**Table 1:** Types of pseudoviral packaging systems and their applications.

Pseudoviral Packaging System	Vector Virus	Research Area and Application
Lentiviral vector packaging systems	HIV-1, SIV, FIV	Mechanism of virus entry, evaluation of neutralization antibody, screen of antiviral drugs, vaccine development, gene therapy, NAT, etc.
VSV packaging system	VSV	Mechanism of virus entry, function of glycoprotein, neutralization antibody assay, antiviral drug screening, vaccine development, etc.
MLV packaging system	MLV	Mechanism of virus entry, interaction of virus and host cell, evaluation of neutralization antibody, etc.
AdV and AAV packaging systems	AdV, AAV	Gene therapy, tumor immunotherapy, vaccine development
Armored RNA packaging system	MS2 bacteriophage	Act as reference material in NAT
Other packaging systems	alphavirus, flavivirus, picornavirus, paramyxovirus, etc.	Mechanism of virus entry and reproduction, vaccine research, antiviral compounds screening, etc.

## Abbreviations

The following abbreviations are used in this manuscript:

NAT	Nucleic acid testing
RM	Reference material
COVID-2019	Coronavirus disease-2019
SARS-CoV-2	Severe acute respiratory syndrome coronavirus 2
EBOV	Ebola virus
RSV	Respiratory syncytial virus
MERS-CoV	Middle East respiratory syndrome virus
ELISA	Enzyme-linked immunosorbent assays
PCR	Polymerase Chain Reaction
EQA	External quality assessment
WHO	World Health Organization
NIBSC	National Institute for Biological Standards and Control
HCV	Hepatitis C virus
NIM	National Institute of Metrology
CRMs	Certified reference materials
naRM	Nucleic acid reference material
ISO	International Organization for Standardization
BSL	Biosafety Level
HIV-1	Human immunodeficiency virus type 1
SIV	Simian immunodeficiency virus
FIV	Feline immunodeficiency virus
CMV	Cytomegalovirus
DENV	Dengue virus
VSV	Vesicular stomatitis virus
MLV	Murine leukemia virus
MMLV	Monkey murine leukemia virus
LTR	Long terminal repeats
AdV	Adenovirus
AAV	Adeno-associated virus
*E. coli*	*Escherichia coli*
VLPs	Virus-like particles
LoD	Limits of detection
POCT	Point-of-care testing
AI	Artificial intelligence
qPCR	Quantitative PCR
dPCR	Digital PCR
CV	Coefficient of variation
PBS	Phosphate-buffered saline
IBC	Institutional Biosafety Committee
